# The role of theory-driven graphic warning labels in motivation to quit: a qualitative study on perceptions from low-income, urban smokers

**DOI:** 10.1186/s12889-015-1438-6

**Published:** 2015-02-07

**Authors:** Erin L Mead, Joanna E Cohen, Caitlin E Kennedy, Joseph Gallo, Carl A Latkin

**Affiliations:** Department of Health, Behavior and Society, Johns Hopkins University Bloomberg School of Public Health, Baltimore, MD USA; Tobacco Center of Regulatory Science, Department of Behavioral and Community Health, University of Maryland School of Public Health, College Park, MD 20742-2611 USA; Department of Health, Behavior and Society, Institute for Global Tobacco Control, Johns Hopkins University Bloomberg School of Public Health, Baltimore, MD USA; Department of International Health, Johns Hopkins University Bloomberg School of Public Health, Baltimore, MD USA; Department of Mental Health, Johns Hopkins University Bloomberg School of Public Health, Baltimore, Maryland USA

**Keywords:** Graphic warning labels, Health communication, Behavioral theories, Smoking cessation, Motivation, Low-income adults, Qualitative research

## Abstract

**Background:**

Use of communication theories in the development of pictorial health warning labels (graphic warning labels) for cigarette packaging might enhance labels’ impact on motivation to quit, but research has been limited, particularly among low socioeconomic status (SES) populations in the U.S. This qualitative study explored perceptions of theory-based graphic warning labels and their role in motivation to quit among low-income smokers.

**Methods:**

A cross-sectional qualitative study was conducted with 25 low-income adult smokers in Baltimore, Maryland, who were purposively sampled from a community-based source population. Semi-structured, in-depth interviews were conducted from January to February 2014. Participants were asked about the motivational impact of 12 labels falling into four content categories: negative depictions of the health effects of smoking to smokers and others, and positive depictions of the benefits of quitting to smokers and others. Data were coded using a combined inductive/deductive approach and analyzed thematically through framework analysis.

**Results:**

Labels depicting negative health effects to smokers were identified as most motivational, followed by labels depicting negative health effects to others. Reasons included perceived severity of and susceptibility to the effects, negative emotional reactions (such as fear), and concern for children. Labels about the benefits of quitting were described as motivational because of their hopefulness, characters as role models, and desire to improve family health. Reasons why labels were described as not motivational included lack of impact on perceived severity/susceptibility, low credibility, and fatalistic attitudes regarding the inevitability of disease.

**Conclusions:**

Labels designed to increase risk perceptions from smoking might be significant sources of motivation for low SES smokers. Findings suggest innovative theory-driven approaches for the design of labels, such as using former smokers as role models, contrasting healthy and unhealthy characters, and socially-oriented labels, might motivate low SES smokers to quit.

## Background

Globally, the U.S. ranks among the highest in income inequality among high-income countries [1], and income inequality continues to grow [2]. The association between socioeconomic inequality and increased mortality can be partially explained by greater prevalence of risk behaviors, including smoking [3,4]. In the U.S., low socioeconomic status (SES) smokers are less likely to attempt to quit and achieve cessation for ≥6 months compared to higher SES smokers [5,6]. Smoking prevalence is highest in low SES neighborhoods, which may be due to targeted marketing [7-9] and the use of smoking as a coping mechanism for stress and as a shared behavior that fosters norms favorable toward smoking and significant barriers to quitting [10,11]. In Baltimore, Maryland, for example, smoking prevalence is 58% in some low SES neighborhoods [12]. Moreover, the global fiscal crisis of 2008 contributed to increased smoking prevalence among the unemployed in the U.S., thus increasing health inequities [13]. Public health interventions that effectively promote cessation among low SES smokers are needed to help close the health equity gap, particularly in light of the growing socioeconomic inequality in the U.S.

Research has shown that motivation to quit is associated with making quit attempts [14-16]. The term motivation conveys both an emotional component and a rational, cognitive component that weighs the benefits and risks of changing behavior [14]. Individuals can be intrinsically motivated—that is, by inherent desires to achieve internal rewards, such as improved health or enjoyment—and extrinsically motivated—that is, by desires to achieve rewards or avoid punishments from external sources, including social influences [17,18]. One approach to increase motivation and change behavior is theorized by the extended parallel process model, which posits that individuals are motivated to act through fear if they perceive a high level of risk from their engagement in an unhealthy behavior, specifically that they are susceptible to severe, negative consequences [19]. If they believe that they have the ability to change their behavior (perceived self-efficacy) and the behavioral change will reduce their risk of negative outcomes (perceived response efficacy), they are motivated to engage in the healthier behavior. According to this theory, then, tobacco control messaging that aims to increase smokers’ motivation to quit should contain both threat and efficacy messages to increase risk perceptions and efficacy beliefs.

One policy approach used by many countries to motivate cessation is graphic warning labels, which are pictorial health warning labels on cigarette packaging describing the dangers of tobacco use [20,21]. Labels have largely relied on fear appeals to increase smokers’ risk perceptions using depictions of the negative effects of smoking [20]. Studies in several countries have examined the vividness of pictures and the portrayal of internal vs. external health effects [20], but little work has explicitly compared messages about the effects to others to messages about the effects to smokers [22]. Labels portraying the effects to others may be important given that social concern for others is a significant motivating factor for smokers to quit [23]. They may also provide a source of extrinsic motivation to quit smoking by invoking social influence. Moreover, limited research on labels’ influence on efficacy beliefs has found very little impact, likely due to the lack of theory-driven efficacy messages [24-26].

Exploring the role of graphic warning labels on motivation to quit among low SES smokers might be particularly important given the evidence of a health knowledge gap [27,28]. According to the health knowledge gap hypothesis, high SES individuals are able to more easily and rapidly obtain health information than low SES individuals, and this gap is linked to health disparities [27-29]. Although research has shown that graphic warning labels are more effective than text-only labels regardless of SES [30], the effectiveness of specific types of content (such as testimonial vs. didactic information) might differ by socioeconomic factors [31,32].

As a first step to address these gaps in the literature, this qualitative study explored perceptions of graphic warning labels and their influence on motivation to quit among low-income, urban smokers. To examine what content might play a bigger role in motivation, we developed and compared theory-based labels that varied based on: depictions of the effects of smoking or quitting to smokers and others, level of threat from smoking, and efficacy messages.

## Methods

From January to February 2014, semi-structured in-depth interviews were conducted with 25 low-income smokers who had participated in a quantitative survey on tobacco use, attitudes and communication channels in Baltimore, Maryland. Inclusion criteria were aged ≥18 years, smoked ≥100 cigarettes in lifetime, and smoked cigarettes in the past 30 days at the time of the quantitative study. We purposively sampled from the pool of survey participants for an adequate distribution by gender and age group (18–39 and ≥40 years) to capture variations among younger and older middle-aged smokers who may have different health concerns. All individuals who were approached agreed to participate in this qualitative study.

Recruitment for the survey occurred in low-income neighborhoods through street outreach and word-of-mouth by trained staff from the Lighthouse Studies at Peer Point, a community-based research center that works with low SES populations with a high prevalence of injection drug use and HIV [33]. This population was chosen because of its high smoking prevalence and significant barriers to cessation; unpublished data from three studies showed smoking rates of 83-88%.

### Procedures

Twelve graphic warning labels were developed using (whenever possible) the warning statements mandated by U.S. law and either existing labels (from the U.S., Canada, Brazil, and Australia) or pictures (Table 1). Labels also included the U.S. Quitline number. The labels fell into one of four content categories: negative depiction of the health effects of smoking to the smoker (n = 4) and others (child or adult non-smoker; n = 4), and a positive message about quitting for the smoker (n = 2) and others (n = 2).

**Table 1:** **Characteristics of the graphic warning labels**

**Label #**	**Label image**	**Content category**	**Origin**
**1**		Negative consequences of smoking to smokers	Adapted from Canada, 2012
**2**		Negative consequences of smoking to smokers	Adapted from Brazil, 2009 [34]
**3**		Negative consequences of smoking to smokers	Adapted from Canada, 2012 [35]
**4**		Negative consequences of smoking to smokers	Adapted from U.S., 2012 (proposed) [36]
**5**		Negative consequences of smoking to others^1^	Adapted from Australia, 2011 [37]
**6**		Negative consequences of smoking to others^1^	Adapted from U.S., 2012 (proposed) [36]
**7**		Negative consequences of smoking to others^1^	Adapted from U.S., 2012 (proposed) [36]
**8**		Negative consequences of smoking to others^1^	Adapted from Brazil, 2009 [34]
**9**		Benefits of quitting for smokers	Created using purchased image, 2014
**10**		Benefits of quitting for smokers	Adapted from U.S., 2012 (proposed) [36]
**11**		Benefits of quitting for others^1^	Created using purchased image, 2014
**12**		Benefits of quitting for others^1^	Created using purchased image, 2014

^1^Others include infants, children and adult non-smokers.

The labels were designed to portray different levels of threat and convey efficacy messages following the extended parallel process model and social cognitive theory [19,38]. Based on previous categorization [22], labels with a vivid picture of the negative effects were categorized as high threat, nonvivid picture of the negative effects as low threat, and positive picture about quitting as no threat. Following social cognitive theory [38], the efficacy messages were in text format placed to the right of the picture and designed to increase self-efficacy to quit (confidence in ability to quit successfully), response efficacy of quitting (effectiveness of quitting on improving health), and response efficacy of the Quitline (effectiveness of the Quitline to aid in cessation). The labels and interview guide were developed and finalized through pilot testing with five participants and Lighthouse staff.

Participants were asked about their cognitive and affective reactions to each label (such as what was the main message of the label and how it made them feel) and which labels were most likely to motivate them to quit. Data on age, gender, race, marital status, educational level, employment status, income, smoking, and quitting behavior were also collected. The Johns Hopkins Bloomberg School of Public Health Institutional Review Board approved this study. Interviews took place in a private office at the Lighthouse, were audio-recorded, and lasted 1–2 hours. Participants provided written informed consent and were compensated $25. The lead author (ELM), who is trained in qualitative interviewing and analysis, conducted all recruitment, interviews, and data analysis.

### Data analysis

Transcripts were analyzed using the framework method, a type of thematic analysis using a matrix structure to systematically reduce qualitative data [39]. The lead author developed the coding scheme using a deductive and inductive approach based on the interview guide as well as emerging themes from the data and with input from two co-authors (CEK and JEC). The lead author conducted analytic memoing to reflect on emerging themes and issues, including deviant cases. Codes were then grouped into broader categories, such as a category for codes related to motivation to quit. Next, the data were charted into the framework matrix to provide accurate summaries by participant, category, and label. For example, responses were summarized for all codes within the motivation category for each participant and label. Broader themes were developed by comparing codes and categories within and across cases with special attention to deviant cases. The framework approach and matrix structure allowed for the data to be kept within the rich context of each case, facilitated the identification of patterns, and included references to specific transcript lines, thus enhancing rigor and transparency [39]. This study adheres to the RATS guidelines for reporting qualitative studies (http://www.biomedcentral.com/authors/rats).

## Results

Twelve men and 13 women participated and were on average 45 years old (Table 2). Most were African American (n = 22) and earned less than $10,000 in the previous year (n = 16). Many had not completed high school (n = 12). Most reported smoking everyday (n = 23), and many smoked less than one pack per day (n = 11). Fourteen participants reported that they had ever tried to quit, and many had made at least one attempt in the previous 12 months (n = 11) and were currently trying to quit (n = 8).

**Table 2:** **Characteristics of the interview participants (N=25)**

**Characteristics**	**n (%)** ^**1**^
Age in years (mean ± standard deviation)	45 ± 11
Age range in years	22 – 61
Age group	
< 40 years	10 (40)
≥ 40 years	15 (60)
Race	
African American	22 (88)
Caucasian	3 (12)
Gender	
Male	12 (48)
Female	13 (52)
Marital status	
Single	12 (48)
Married/partnered	12 (48)
Separated	1 (4)
Level of education	
Less than high school	12 (48)
High school or GED^2^ completed	11 (44)
Some college, college completed or higher	2 (8)
Employment status	
Employed full time	1 (4)
Unemployed	7 (28)
Unable to work or retired	16 (64)
Student	1 (4)
Personal pre-tax income from previous year	
Less than $10,000	16 (64)
$10,000 – 29,999	6 (24)
$30,000 – 49,999	1 (4)
Not applicable	2 (8)
Smoking frequency	
Once a week or a few times a week	2 (8)
Everyday	23 (92)
Cigarette packs smoked per day^3^	
Less than 1 pack	11 (44)
1 pack	7 (28)
More than 1 pack	7 (28)
Ever tried to quit	14 (56)
≥1 quit attempt in the previous 12 months^4^	11 (79)
Currently trying to quit^4^	8 (57)

^1^Frequency and percentage reported unless otherwise noted.

^2^General Educational Development (GED).

^3^On days that they smoked.

^4^Only among participants who reported ever trying to quit (n=14).

### Role in motivation to quit

Participants were asked about the labels’ influence on their motivation to quit. They most often identified labels depicting the negative consequences of smoking to smokers, regardless of whether the label portrayed high or low threat, because of the influence on their risk perceptions (perceived severity and susceptibility):

Because you look at which way you going… You going to [get] a messed up heart and you going to your throat cancer or whatever he got. And oh, my God, that [label #2] speak for itself. That one speak for itself. (man, 47 years).

I have to say, this kinda changes your mind, but after going outside smoking a cigarette,… you won’t be enjoying it as much after this… I mean, you know it’s harming you, but you don’t know it’s harming [you] to this [extent]… [When] you actually see a heart like this, you just be like, “Wow”. You know what I’m saying? It really makes you think. (man, 31 years).

As illustrated by these quotes, participants were motivated by the severity of the health effects portrayed and how shocking they can be when presented as a picture. Moreover, the labels made them worry about what smoking was doing to their bodies and whether they would experience these conditions in the future. Some participants also reported feeling scared, illustrating the influence of negative affective reactions on motivation. The label reported by the most participants as motivational (label #2, Table 1) provided new information about the effects of smoking. The new information combined with the high threat picture was highly motivational, even for those who found most of the labels unmotivating.

After the labels depicting risks to smokers, participants most often identified the labels depicting negative effects to others as motivational, because these labels showed a health effect they thought was severe and to which others were highly susceptible. Labels depicting children were more motivating than those depicting adults. Participants described a general moral need to protect helpless children:

Oh, very motivating. You don’t want to hurt your kids, no one wants to hurt their kids, that’s very motivating… I don’t have kids, but if I did,… I would much rather… do something for them as opposed to doing it for myself. (woman, 36 years).

Several younger men and women, many of whom did not have children themselves, were particularly affected by these labels and expressed concern about their future children’s health and the need to quit to have healthy babies.

For men and women who had children, grandchildren, nieces, and nephews, these labels made them concerned about their health: “My grandson have asthma real bad and he was hospitalized three times. So that made me pick [these labels]… I want to be around, healthy. I want to see my grandchildren graduate from school, get married or whatever” (woman, 52 years). Participants were not only concerned about the effect of secondhand smoke on the children, but also felt concern for their own health and a desire to live longer for the children. Several participants described these labels as encouraging them to think about how their future poor health and premature death would negatively impact their families emotionally: “Sometimes it’s too late and then you actually see more pain from them than you actually going through” (woman, 22 years).

Different patterns emerged by participants’ quitting behaviors. Most participants who were currently trying to quit or had never tried to quit identified both types of negative labels (effects to smokers and others) as motivational. In contrast, participants who were not currently trying to quit but had tried in the past were only motivated by the labels depicting their own risk.

Overall, participants were more motivated by the negative labels than the positive labels about the benefits of quitting. However, several participants said the positive labels were very motivational, with a relatively equal mixture of people motivated by the benefits of quitting for smokers, others, and both. Participants stated these labels were motivational because they showed hopeful messages about people who quit and the benefits of quitting. They viewed some characters as role models for quitting and its benefits. In addition, participants reported that quitting for others was a highly motivational message and discussed their family members as an inspiration:

[My husband’s] the one with the secondhand smoke. And I know I love him so much – that’s why I been trying to cut down… I don’t want to… [make] his health bad because of my smoking. So I’m really thinking. (woman, 46 years).

Notably, none of the positive labels were motivating to participants who had made a quit attempt, but they were motivating to participants currently trying to quit or who had never tried to quit.

### Factors inhibiting role in motivation

Participants also described why the labels failed to motivate them and why the labels might fail to motivate others. The most significant factor discussed was that the labels failed to influence their perceived severity and susceptibility. For several participants, the positive labels about the benefits of quitting did not depict serious health conditions, which they considered necessary for motivation. When discussing the negative labels about the consequences of smoking, several participants indicated that smokers think they are unlikely to get the health conditions:

Even smoking, drinking, whatever, drugs, whatever they are doing, [older people] tend to think that if I stop now, all these ailments are going to come up all of a sudden. So I don’t think the picture would really affect a lot of people if they been smoking for a long period of time because they think, ‘I’ve been smoking all this time and nothing happened yet.’ (woman, 52 years).

Another inhibiting factor was that some participants doubted the labels’ credibility. They questioned the credibility of the characters pictured on the positive labels, saying, for example, that they were actors or real people who did not actually quit. A few participants also doubted the validity of the text, distrusting that smoking caused the health conditions or that quitting would improve health.

Even when believed, several participants reported that improving health and avoiding disease was not sufficient motivation and they could still fall sick. As one older man (aged 60 years) said, “It’s not an ad that I would adhere to… It’s like I’ve always thought: if you’re going to get [a disease], you’re going to get it; if you’re not, you’re not.” As described by another older man (aged 55 years), this fatalism could affect multiple aspects of individuals’ lives: “A lot of people just don't… want to try to better themselves. You got some people that just don't want to quit. Doesn't matter. ‘Whatever's going to happen is going to happen’”. For these participants, the health threat portrayed by the labels was not motivational.

Some participants also described labels as unmotivating because the characters did not have inspirational stories or did not correspond to the text, they had no desire to emulate the characters who quit, and the characters were not similar to them. For the labels about quitting for others, some participants said these labels did not apply to them because they did not currently have children (or did not plan to have future children) and were not family-oriented people.

Three participants reported that none of the labels were motivational, and each may represent different subsets of the smoker population. An older man (aged 61 years) described a high level of intrinsic motivation to quit, such that the labels provided very little extrinsic motivation. He was motivated to quit to improve his health and had reduced his smoking. He was somewhat motivated by the positive labels because he wanted to look healthy like the characters and stated quitting for others was a good message. A younger man (aged 39 years), who had never made a quit attempt, also expressed a lot of concern about his health and was motivated to quit both to be healthier and make his family happy. However, he said he was unable to overcome his nicotine addiction – in other words, he had low self-efficacy to quit. He discussed how the labels made him think about quitting sooner, but could not motivate him to quit at the moment.

Lastly, a younger woman (aged 39 years) was not motivated by the labels because she had no desire to quit. She expressed a somewhat fatalistic attitude as well as low perceived risk from smoking, describing doubts that smoking would kill her and, if it does, “so be it”. She was initially somewhat motivated by label #11 because it showed quitting as a family activity, but then became distrustful of the characters’ credibility. Overall, she was accepting of her decision to smoke and what it might lead to: “Because I’m at the point in my life that I’m going to do what I want to do, and I already know what I’m doing to myself and I got to live with that. That’s the truth I decide in me. That’s the truth I got to live with”. This quote illustrates the limited impact that labels may have on smokers with little desire to quit.

## Discussion

This qualitative study contributes to the graphic warning label literature by exploring low-income, urban U.S. smokers’ perceptions of theory-based labels and the labels’ role in their motivation to quit smoking. We found that participants were most motivated by labels portraying the negative consequences of smoking (negative labels), especially consequences to smokers; high and low threat labels were both motivational. The threat portrayed in a message—characterized by severity of and susceptibility to health conditions—intrinsically motivates action through fear and by increasing individuals’ perceptions of their risk [19]. Indeed, we found that perceived severity, feelings of susceptibility, and negative emotional reactions—such as fear and concern for others—were major reasons why participants were motivated by the negative labels. These findings are consistent with other research showing that vivid depictions of negative effects promote cessation-related attitudes and behaviors [20,22].

Some negative labels failed to motivate participants because of low perceived susceptibility. Research has shown that smokers have an optimistic bias regarding their cancer risk compared to nonsmokers and other smokers [40], and increasing perceived vulnerability can increase motivation to quit [41]. Vivid pictures that convey high threat, depictions of conditions commonly experienced by smokers with a progression to more serious outcomes, and use of characters who are highly similar to smokers and portrayed as susceptible to conditions are some ways in which labels could be designed to enhance perceived susceptibility.

Participants were also motivated by the positive labels about the benefits of quitting because of their hopefulness, use of characters as role models, and depiction of the benefits for their and their families’ health and emotional wellbeing. These findings illustrate the potential for self-efficacy and response efficacy messages on labels to motivate quitting. Our prior work showed that participants vicariously experienced characters’ quit successes portrayed on the labels, and these experiences played a role in their self-efficacy beliefs [Mead, Cohen, Kennedy, Gallo and Latkin: The influence of graphic warning labels on efficacy beliefs and risk perceptions: a qualitative study with low-income, urban smokers, submitted]. Using a narrative format to enhance vicarious experiences and overcome message resistance [42], labels can share the testimonials of ex-smokers who were able to quit and potentially increase self-efficacy and motivation. However, formative research is needed to develop realistic models to avoid doubts about their credibility. To increase motivation through response efficacy messages, labels can describe how quitting reduces risk to promote message acceptance. To address the critique that positive labels did not portray a significant threat, labels could show someone whose health improved after quitting (on the back of the pack) contrasted with someone who did not quit and experienced deteriorating health (on the front of the pack).

Notably, participants who had made a quit attempt were most motivated by labels about their own risks of smoking, rather than risks to others, and not motivated by positive labels. Individuals at different stages of the process towards behavior change are motivated by different factors [43]. For example, smokers who are not ready to quit may be motivated by messages providing new information and allowing them to experience negative emotions about smoking. Indeed, participants reported these factors as motivating characteristics of the labels. Using stages of change theory and audience segmentation techniques [43], labels can be designed to target smokers by readiness to quit, including those who are seemingly unmotivated to quit, such as helping smokers weigh the pros and cons of quitting.

Socially-oriented messages are an untapped potential avenue for future messaging. Our finding that smokers who never tried to quit were motivated by messages about effects to others is consistent with other evidence showing they are more likely to attempt to quit if they perceive that others desire them to quit [15]. Labels can utilize this social concern to provide extrinsic motivation for quitting and better target smokers who have never made a quit attempt. For some smokers, social concern may be the only motivational label message, as exemplified by the younger woman who was partly motivated by a label about family support for quitting.

“Fatalistic” attitudes regarding health were present in a subset of participants. Contrary to the extended parallel process model, threat messages did not appear to be sufficient to motivate action in this group. The participants live within economically deprived areas in which smoking might be considered lower risk than other risks, such as injection drug use, HIV, and violence. When examining labels’ effectiveness, future work should consider such attitudes and contextual factors that may influence the impact of labels. Research is needed to examine what factors other than risk perceptions might motivate smokers holding fatalistic attitudes.

There are several strengths and limitations to this study. We used purposive sampling of smokers who were recruited from low-income, urban neighborhoods. Although the community-based, low-income, predominantly African-American sample allowed for the participation of an understudied population, the transferability of the findings to other populations might be limited. However, the findings might be relevant beyond the population studied, including populations in other countries, because evidence-based theories were used in the design and analysis of labels. For example, future work might test socially-oriented labels among cultures that are less individualistic and more community-oriented. High SES smokers were not included because the focus of the study was on low SES populations as there has been insufficient research on persons with low SES and smoking. The qualitative methodology allowed for an in-depth exploration of smokers’ perceptions of the motivational aspects of labels, but the cross-sectional design precludes conclusions about the causal relationship between labels and smokers’ motivations and behaviors.

## Conclusions

The role of graphic warning labels in risk perceptions, self-efficacy beliefs, and response efficacy beliefs were reported as motivators for cessation in this population of low-income, urban smokers. Our findings suggest multiple avenues for the design of future labels that might promote smoking cessation. Labels portraying negative effects of smoking, socially-oriented messages, and benefits of quitting are potential approaches to motivate cessation. Differences in the perceptions of labels that emerged by quit attempt history suggests including warning labels that address smokers’ readiness to quit might enhance effectiveness. Several factors that might influence the impact of labels, such as low perceived susceptibility, quit attempt history, and fatalistic attitudes, should be examined and addressed in future work. By using evidence-based theories to design and study graphic warning labels, these findings are a first step towards promoting smoking cessation among low SES and minority populations through graphic warning labels.
